# Comparative Study of the Effective Properties of 0–3 and Gyroid Triply Periodic Minimal Surface Cement‐Piezocomposites

**DOI:** 10.1002/gch2.202200122

**Published:** 2022-11-29

**Authors:** Saptarshi Karmakar, Raj Kiran, Rahul Vaish, Vishal Singh Chauhan, Samia Ben Ahmed, Imed Boukhris, Wonseop Hwang, Tae Hyun Sung, Anuruddh Kumar

**Affiliations:** ^1^ School of Mechanical and Materials Engineering Indian Institute of Technology Mandi Himachal Pradesh 175075 India; ^2^ School of Mechanical and Aerospace Engineering Nanyang Technological University 50 Nanyang Avenue Singapore 639798 Singapore; ^3^ Departement of Chemistry College of Sciences King Khalid University Abha P.O. Box 9004 Saudi Arabia; ^4^ Department of Physics Faculty of Science King Khalid University Abha P.O. Box 9004 Saudi Arabia; ^5^ Laboratoire des matériaux composites céramiques et polymères (LaMaCoP) Département de Physique Faculté des sciences de Sfax BP 805 Université de Sfax Sfax 3000 Tunisie; ^6^ Department of Electrical Engineering Hanyang University 222, Wangsimni‐ro Seongdong‐gu Seoul 04763 Korea; ^7^ Center for Creative Convergence Education Hanyang University 222, Wangsimni‐ro Seongdong‐gu Seoul 04763 Korea

**Keywords:** cement matrix, lead‐free piezoelectric materials, piezocomposites, TPMS

## Abstract

In the present numerical simulation work, effective elastic and piezoelectric properties are calculated and a comparative study is conducted on a cement matrix‐based piezocomposite with 0–3 and gyroid triply periodic minimal surface (TPMS) inclusions. The present study compares the effective properties of different piezoelectric materials having two different types of connectivity of the inclusions namely, 0–3 inclusions where the inclusions are physically separated from each other and are embedded within the matrix and the second one is TPMS inclusions having interpenetrating phase type connectivity. Effective properties are calculated for four different materials at five different volume fractions namely, 10%, 15%, 20%, 25%, and 30% volume fractions of inclusion by volume. In terms of effective properties and direct piezoelectric effect, TPMS piezocomposite is found to perform better compared to 0–3 piezocomposite. Lead‐free piezoelectric material 0.5Ba(Ca_0.8_Zr_0.2_)O_3_ − 0.5(Ba_0.7_Ca_0.3_)TiO_3_ demonstrates better performance compared to all other material inclusions studied. The present study attempts to highlight improved piezoelectric effective properties of lead‐free material‐based piezocomposites with TPMS inclusions.

## Graphical abstract

A comparative study conducted on 0–3 and triply periodic minimal surface (TPMS) piezocomposites is presented. Lead‐free barium calcium zirconium titanate (BCZT) TPMS piezocomposite demonstrates about 50 and 33 times increment in *e*_31_ and *e*_33_ compared to 0–3. Gyroid demonstrates better stiffness and generates 2.5 times more voltage in response to the direct piezoelectric effect than 0–3 piezocomposite for BCZT material.

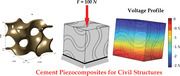

## 1 Introduction

Materials that generate a voltage output when an external load is applied are popularly known as piezoelectric materials and the corresponding effect is often referred to as the piezoelectric effect. The piezoelectric effect makes these materials suitable for applications involving sensors. Though piezoelectric ceramics are superior to piezoceramics and piezopolymers in terms of piezoelectric properties, certain drawbacks like rigidity, brittleness, etc., make them a second choice compared to other forms of piezoelectric materials like polymers and polymer composites. Piezoelectric polymer composites composed of piezoelectric ceramic inclusions and polymer matrix are becoming quite popular and are receiving substantial attention from the research community these days and are often the preferred choices for applications like sensing and energy harvesting.^[^ ^1^ ^]^ The most common way of utilizing this material for sensing applications is to attach them as a patch to a host vibrating structure. The voltage generated from vibrations is often used for sensing applications. The above‐mentioned piezoelectric phenomenon can also be applied to buildings and structures to monitor their health and structural integrity and are essential for civil infrastructures of critical importance like dams, bridges, nuclear reactors, etc.

Attempts have already been made to integrate sensing capabilities into civil engineering structures to make them smart for health monitoring. Sensing capabilities integrated into such structures have proven to be a very useful technique to monitor the health of such structures. Moreover, as concrete structures are prone to age degradation, it becomes even more important to devise novel ways to monitor their health over time. The common ways in which piezoelectric materials are used as sensors and actuators involve attaching a piezoelectric patch material to a piezoelectric host structure subjected to vibration or actuation. But such modes of operation may not be suitable in the case of concrete‐based material due to inherent differences in material properties and volume stability.^[^ ^2^, ^3^ ^]^ This motivated the development of cement‐based piezo composites to serve structural health monitoring purposes. Different cement matrix‐based piezo composites like 0–3 and 2–2 with lead zirconium titanate (PZT) piezoelectric material have already been reported in the literature.^[^ ^2^, ^4^, ^5^ ^]^

Experimental results demonstrated compatibility between cement and piezoelectric inclusions and the retention of piezoelectric properties for sensor applications ensuring the potential use of such composites for sensor applications.^[^ ^6^ ^]^ Several researchers have verified and improved the compatibility of piezoelectric inclusions within cement matrices.^[^ ^2^, ^3^, ^5^, ^6^ ^]^ While piezoelectric ceramics are characterized by their electromechanical parameters, for piezoelectric composites, the corresponding effective values of the parameters are used to characterize them. Both experimental and theoretical methods can be used to characterize a piezoelectric composite material.^[^ ^4^, ^7^, ^8^, ^9^ ^]^ Piezocomposites are often classified based on the connectivity of the inclusions within the matrix material. Common connectivity patterns reported in literature are 0–0, 0–1, 0–2, ⋅⋅⋅,3–3, etc.^[^ ^6^ ^]^ The connectivity between the matrix and the inclusions controls the patterns of the electrical flux lines and the mechanical properties of the composite, which, in turn, decides the overall performance of the piezoelectric composite.^[^ ^10^, ^11^, ^12^ ^]^ Perhaps the most complicated and interesting connectivity pattern is the 3–3 connectivity pattern where the matrix and the inclusion phases create a 3D network such that the two phases interpenetrate. In the 3–3 connectivity pattern the piezoelectric inclusions have electrical continuity in three dimensions due to which they can produce a large piezoelectric effect. Recent developments in manufacturing and 3D printing capabilities have allowed the fabrication of complex mathematically defined surfaces. Recently geometries with minimal surfaces have been reported in the literature and referred to as triply periodic minimal surfaces (TPMSs).^[^ ^13^, ^14^, ^15^, ^16^ ^]^ The mean curvature of these surfaces is zero at all points on the surface. These surfaces can also have infinite periodicity and can extend in three dimensions forming interpenetrating composites having 3–3 connectivity thus improving their effective properties.^[^ ^17^, ^18^, ^19^ ^]^ TPMS has been reported to improve and enhance various mechanical properties like shear and elastic modulus etc.^[^ ^13^, ^20^, ^21^, ^22^ ^]^ Xu et al. reported a TPMS‐based piezocomposite with improved piezoelectric performance. Though the authors could find several pieces of literature reporting improved piezoelectric performance of different TPMS‐based piezocomposites, not much work was found in the literature that involve cement matrix and TPMS inclusion‐based piezocomposite. This has motivated the authors to explore cement and TPMS‐based piezocomposite which could be used for sensing applications in civil infrastructures. Different TPMS structures have been reported in the literature like Neovius, gyroid, primitive, etc. These TPMS structures are characterized by specific mathematical equations which decide their shape and the parameters in those equations decides the volume fractions.

The present study is a numerical simulation‐based study that compares the effective elastic and piezoelectric parameters of a 0–3 and TPMS inclusion‐based piezocomposites with cement as matrix material which could be used in civil structures for sensing applications. Among various TPMS structures, the gyroid‐based structure has been considered in the present study because of its ability to undergo large strain before failure. Moreover, the gyroid structures deform in a bending‐dominated manner which makes them suitable for sensing applications.^[^ ^23^ ^]^ Different piezocomposites with Portland cement matrix and four different piezoelectric material inclusions were considered for the present study.

Four different piezoelectric materials were considered, three lead‐free and one lead‐based namely, 0.5Ba(Ca_0.8_Zr_0.2_)O_3_ − 0.5(Ba_0.7_Ca_0.3_)TiO_3_ (BCZT), Li_0.03_(K_0.48_Na_0.52_)_0.97_(Nb_0.8_Ta_0.2_)O_3_ (KNNLT), 0.965(K_0.48_Na_0.52_)(Nb_0.96_Sb_0.04_)O_3_ − 0.035(Bi_0.5_Na_0.5_)Zr_0.15_Hf_0.75_O_3_ (KNNS − BNZH), and (Zn_1/3_Nb_2/3_)O_3_ − (6 − 7%)PbTiO_3_ (PZN − PT).

The present study attempts to explore different lead‐free piezoelectric material‐based cement piezo composites which can be applied to civil‐based structures for health monitoring. Lead‐free piezoelectric material has been explored before, but mostly in the context of sensors and actuator applications.^[^ ^24^, ^25^, ^26^ ^]^ However, to the best of the author's knowledge and understanding, not much literature is available on cement piezocomposite using lead‐free piezoelectric materials and specifically with TPMS‐based inclusions. Enhanced properties of TPMS‐based composites have already been reported in the literature.^[^ ^27^, ^28^, ^29^, ^30^ ^]^ In the past several years’ significant research has been conducted on smart multifunctional concrete structures built for very specific and dedicated purposes.^[^ ^31^, ^32^, ^33^, ^34^, ^35^, ^36^, ^37^, ^38^, ^39^, ^40^, ^41^, ^42^, ^43^, ^44^, ^45^, ^46^, ^47^, ^48^, ^49^ ^]^ This has motivated the authors to explore cement‐TPMS‐based piezocomposites, specifically keeping in mind the self‐sensing and energy‐harvesting applications of concrete structures.^[^ ^35^, ^49^, ^50^, ^51^, ^52^ ^]^ The present study can be significant considering the use of cement‐TPMS‐based piezocomposites with enhanced properties in the context of structural health monitoring of critical structures like dams, nuclear reactors, high‐rise buildings, bridges, etc.

The following Section 2 in this paper will discuss different piezoelectric materials considered for the present study. The next section, describes the finite element method‐based numerical technique to calculate the effective properties. Finally, a comparative analysis of the effective properties of both the 0–3 and TPMS‐based piezocomposite for all the materials considered in the present study is reported in the result and discussion section.

## 2 Experimental Section

This study deals with a cement‐based piezoelectric composite having four different piezoelectric materials namely, BCZT, KNNLT, KNNS–BNZH, and PZN–PT. Effective properties were calculated for all the compositions considering both 0–3 and TPMS based piezocomposite at four different volume fractions varying between 10% and 30% in steps of 5%. The inclusion volume fraction for the 0–3 composite was generated using the random sequential adsorption algorithm.^[^ ^53^, ^54^, ^55^ ^]^ This algorithm places the inclusion particles randomly within the representative volume element (RVE) zone and is adsorbed within the system if the particles don't interpenetrate with a previously adsorbed particle. Once a particle is adsorbed, its position remains fixed, and the next particles are randomly placed based on the previously placed particles until the final volume fraction of the inclusion within the RVE is achieved. The inclusion volume fraction and the desirable initial size of the inclusion particles or the desired number of the particles are provided to the algorithm based on which the algorithm starts placing the incisions randomly. For the TPMS inclusions, the volume fraction is adjusted by controlling a constant parameter *t* in the equation of the TPMS geometry discussed further in article §3. Material properties used for the present study were taken from the existing literature and are tabulated in **Table** **1**.

**Table 1:** Properties of piezoelectric materials

Material	$S_{11}^{E}$	$S_{12}^{E}$	$S_{13}^{E}$	$S_{33}^{E}$	$S_{44}^{E}$	$S_{66}^{E}$	*d*_31_	*d*_33_	$\varepsilon_{11}^{T}$	$\varepsilon_{33}^{T}$
BCZT^[^ ^57^ ^]^	9.1	−2.7	−2.9	9.5	2.28	2.36	−297.7	576.4	5778	5778
KNNLT^[^ ^58^ ^]^	12	−3.6	−2.9	11.0	32.0	31.3	−50	174	956	776
KNNS − BNZH^[^ ^59^ ^]^	13.7	−6.3	−5	16.8	43.9	40.0	−140	380	2190	2000
PZN − PT^[^ ^60^ ^]^	32.8	−28.5	−1.2	6.5	390.4	122.6	−35	93	11 000	700

The measurement units to measure stiffness and piezoelectric properties *S_ij_* and *d_ij_* are given in terms of × 10^−12^ m^2^ N^−1^ and pC N^−1^; For the matrix material, Portland cement is considered having Young's modulus *E*  =  1.4 × 10^10^ N m^−2^ and Poisson's ratio ν  =  0.2.^[^ ^9^ ^]^

## 3 Numerical Model to Calculate Effective Properties

Several analytical and numerical tools are available to calculate the effective properties of a piezocomposite material.^[^ ^60^, ^61^, ^62^, ^63^ ^]^ Analytical methods are limited to symmetric geometries and don't take into account the generalized loading condition. Other numerical methods are also reported in the literature like semi‐analytical methods, mean‐field methods, etc., but these often ignore local field fluctuations.^[^ ^64^, ^65^, ^66^, ^67^, ^68^ ^]^ For complex shapes, finite element‐based numerical methods can be applied suitably by sufficiently discretizing the domain due to which the complex shapes can be taken care of more easily compared to other analytical methods. In the present study, the finite element method is applied to a unit cell model, which is a RVE of unit dimensions, to calculate the homogenized effective stiffness and piezoelectric properties by applying suitable periodic boundary conditions.^[^ ^69^, ^70^, ^71^, ^72^ ^]^ RVE is a small material volume extracted from the piezocomposite whose effective properties are the same as the bulk composite. Micromechanical approach is used to calculate the effective properties by the RVE approach using finite element method‐based numerical technique.^[^ ^71^, ^72^, ^73^ ^]^ A similar method has been adopted in the present study to calculate the homogenized effective elastic and piezoelectric properties of the piezocomposite. RVE of the 0–3 composite has been generated by placing random size spherical inclusions distributed randomly within a unit cell of the cement matrix. This is the name of a software and not an acronym. DIGIMAT has been used to generate the RVE. For TPMS‐based piezocomposite, a gyroid TPMS structure has been used in the present study. Numerous techniques to generate a TPMS‐based geometric profile have already been reported in the literature.^[^ ^74^, ^75^ ^]^ However, the level‐set approach is very popular and is commonly used because of its simplicity.^[^ ^76^ ^]^ Trigonometric equations of the form ϕ (*x*, *y*, *z*) =  *t* are level set equations where ϕ are different trigonometric functions.^[^ ^77^ ^]^ The function ϕ(*x*, *y*, *z*) defines different minimal surface depending upon the function ϕ and thickness *t*. The equation of gyroid TPMS is given below in Equation (1).^[^ ^78^ ^]^

(1) $$\begin{array}{c} \sin\left(x\right)\cos\left(y\right)+\sin\left(y\right)\cos\left(z\right)+\sin\left(z\right)\cos\left(x\right)=t \end{array}$$

**Figure 1:**
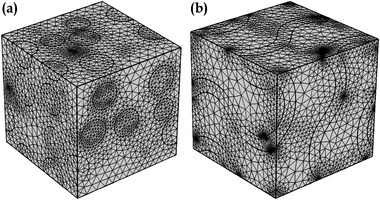
A unit cell model of a) 0–3 composite, b) gyroid TPMS composite.

The RVE volume fraction can be changed by changing the constant value *t* given in Equation (1). A MATLAB‐based standalone package, MSLattice, has been used to generate the gyroid TPMS surface using Equation (1).^[^ ^78^ ^]^ The parameter *t* is calculated by the software itself as per the volume fraction entries feeded into the software package. **Figure** **1**a,b shows the representative volume element for both 0–3 and TPMS piezocomposite discretized sufficiently to obtain a solution. Figure 1a shows the 0–3 composite and Figure 1b shows the gyroid composite where the domain has been discretized using finite element‐based numerical techniques.

**Figure 2:**
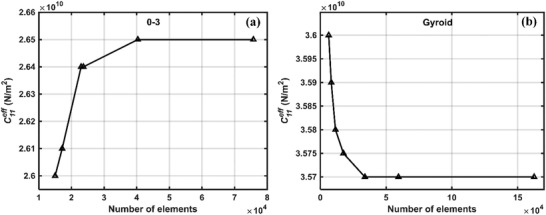
Convergence of solution with increase in number of elements a) 0–3 composite, b) gyroid.

The domains have been discretized with a sufficient number of elements so that any further increase in the number of elements doesn't alter the solution to a great extent. The variation of effective elastic property (stiffness coefficient) $C_{11}^{\text{eff}}$ of BCZT‐cement piezocomposite at 30% volume fraction for a different number of elements are shown in **Figure** **2**.

It can be observed that beyond 4 × 10^4^ and 6 × 10^4^ number of elements in 0–3 and gyroid piezocomposite the solution doesn't vary much. Correspondingly, around 4 × 10^4^ and 6 × 10^4^ number of elements has been used to discretize the domains to find the effective coefficients in the present study. Similar approaches have been adopted for the other volume fractions and other materials.

The piezoelectric effect, both direct and converse, involves both mechanical and piezoelectric parameters which are interdependent, that is, any change in the mechanical property will bring about a change in the piezoelectric properties and vice‐versa. Correspondingly, this type of problem, where the mechanical and electrical properties are interdependent is often referred to as coupled piezoelectric problem.^[^ ^72^ ^]^ The constitutive equation relating the stress parameters to strain parameters using material properties can be written either in stress charge form or in strain charge form. Equation (2) shows the constitutive equation in stress charge form.

(2) $$\begin{array}{c} \left\{\begin{array}{c} T\\ D \end{array}\right\}=\left[\begin{array}{cc} C & -e^{t}\\ e & \varepsilon \end{array}\right]\left\{\begin{array}{c} S\\ E \end{array}\right\} \end{array}$$

The matrix in Equation (2) relates stress *T* and electrical displacement *D* to strain *S* and electric field *E*. Assuming the piezoelectric material to be transversely isotropic, ten material constants are required to relate the above two quantities given in Equation (2). Expanding the above equation, we get Equation (3).

(3) $$\begin{array}{c} \left\{\begin{array}{c} {\bar{T}}_{11}^{}\\ {\bar{T}}_{22}^{}\\ {\bar{T}}_{33}^{}\\ {\bar{T}}_{23}^{}\\ {\bar{T}}_{31}^{}\\ {\bar{T}}_{12}^{}\\ {\bar{D}}_{1}^{}\\ {\bar{D}}_{2}^{}\\ {\bar{D}}_{3}^{} \end{array}\right\}=\left[\begin{array}{ccccccccc} C_{11}^{\text{eff}} & C_{12}^{\text{eff}} & C_{13}^{\text{eff}} & 0 & 0 & 0 & 0 & 0 & -e_{13}^{\text{eff}}\\ C_{12}^{\text{eff}} & C_{11}^{\text{eff}} & C_{13}^{\text{eff}} & 0 & 0 & 0 & 0 & 0 & -e_{13}^{\text{eff}}\\ C_{13}^{\text{eff}} & C_{13}^{\text{eff}} & C_{33}^{\text{eff}} & 0 & 0 & 0 & 0 & 0 & -e_{33}^{\text{eff}}\\ 0 & 0 & 0 & C_{44}^{\text{eff}} & 0 & 0 & 0 & -e_{15}^{\text{eff}} & 0\\ 0 & 0 & 0 & 0 & C_{44}^{\text{eff}} & 0 & -e_{15}^{\text{eff}} & 0 & 0\\ 0 & 0 & 0 & 0 & 0 & C_{66}^{\text{eff}} & 0 & 0 & 0\\ 0 & 0 & 0 & 0 & 0 & 0 & \varepsilon_{11}^{\text{eff}} & 0 & 0\\ 0 & 0 & 0 & 0 & 0 & 0 & 0 & \varepsilon_{11}^{\text{eff}} & 0\\ e_{13}^{\text{eff}} & e_{13}^{\text{eff}} & e_{33}^{\text{eff}} & 0 & 0 & 0 & 0 & 0 & \varepsilon_{33}^{\text{eff}} \end{array}\right]\left\{\begin{array}{c} {\bar{S}}_{11}^{}\\ {\bar{S}}_{22}^{}\\ {\bar{S}}_{33}^{}\\ {\bar{S}}_{23}^{}\\ {\bar{S}}_{31}^{}\\ {\bar{S}}_{12}^{}\\ {\bar{E}}_{1}^{}\\ {\bar{E}}_{2}^{}\\ {\bar{E}}_{3}^{} \end{array}\right\} \end{array}$$

The terms $\bar{T},\;\bar{D},\;\bar{S},$ and $\bar{E}$ used in the above equations are stress, electrical displacement, strain, and electrical field averaged over the entire volume of the RVE. *C* ^eff^, *e* ^eff^, and ε^eff^ are effective values of stiffness coefficient, piezoelectric coefficient, and relative permittivity. A linear piezoelectric response is assumed for the present study.^[^ ^79^ ^]^ A perfect bonding between the inclusion and the cement matrix is assumed. It is also assumed that the piezoelectric inclusions are uniformly poled along the *z* direction. Effective properties are calculated by applying suitable periodic boundary conditions to the RVE. The purpose of applying periodic boundary conditions is to ensure uniform deformation through the entire unit cell volume. Equation (4) expresses the periodic boundary condition.

(4) $$\begin{array}{c} u_{i}={\bar{S}}_{ij}x_{i}+v_{i} \end{array}$$

In the above equation $u_{i},\;{\bar{S}}_{ij}$, and *v_i_* represent displacement, strain, and displacement component's periodic parts. On the two opposite faces, the boundary conditions are written as

(5) $$\begin{array}{c} u_{i}^{K^{+}}={\bar{S}}_{ij}x_{j}^{K^{+}}+v_{i}^{K^{+}} \end{array}$$

(6) $$\begin{array}{c} u_{i}^{K^{-}}={\bar{S}}_{ij}x_{j}^{K^{-}}+v_{i}^{K^{-}} \end{array}$$

In the above equation, *K* denotes a face perpendicular to the axis. *K* ^+^ and *K* ^−^ denote two opposite faces. The macroscopic strain and electric field are calculated by using Equations (7) and (8), respectively.

(7) $$\begin{array}{c} u_{i}^{K^{+}}-u_{i}^{K^{-}}={\bar{S}}_{ij}\left(x_{j}^{K^{+}}-x_{j}^{K^{-}}\right) \end{array}$$

(8) $$\begin{array}{c} \varphi^{K^{+}}-\varphi^{K^{-}}={\bar{E}}_{i}\left(x_{i}^{K^{+}}-x_{i}^{K^{-}}\right) \end{array}$$

Average values are calculated by finding the volume average as given in the equations below

(9) $$\begin{array}{c} {\bar{S}}_{ij}=\frac{1}{V}\text{​}\underset{V}{\int }S_{ij}dV \end{array}$$

(10) $$\begin{array}{c} {\bar{T}}_{ij}=\frac{1}{V}\text{​}\underset{V}{\int }T_{ij}dV \end{array}$$

(11) $$\begin{array}{c} {\bar{E}}_{i}=\frac{1}{V}\text{​}\underset{V}{\int }E_{i}dV \end{array}$$

(12) $$\begin{array}{c} {\bar{D}}_{i}=\frac{1}{V}\text{​}\underset{V}{\int }D_{i}dV \end{array}$$

While applying the boundary condition, all except one component in the strain and displacement field is set to zero value. Correspondingly, the effective properties are calculated using Equation (3). Boundary conditions, calculated using Equation (3), are tabulated in **Table** **2**.

**Table 2:** Boundary conditions to calculate elastic and piezoelectric effective properties

Eff. coeff.	*A* ^−^ (*u_i_*/φ)	*A* ^+^ (*u_i_*/φ)	*B* ^−^ (*u_i_*/φ)	*B* ^+^ (*u_i_*/φ)	*C* ^−^ (*u_i_*/φ)	*C* ^+^ (*u_i_*/φ)	Formula
$C_{11}^{\text{eff}}$	0/ −	*u*_1_/ −	0/ −	0/ −	0/0	0/0	${\bar{T}}_{11}/{\bar{S}}_{11}$
$C_{12}^{\text{eff}}$	0/ −	*u*_1_/ −	0/ −	0/ −	0/0	0/0	${\bar{T}}_{22}/{\bar{S}}_{11}$
$C_{13}^{\text{eff}}$	0/ −	0/ −	0/ −	0/ −	0/0	*u*_3_/ −	${\bar{T}}_{11}/{\bar{S}}_{33}$
$C_{33}^{\text{eff}}$	0/ −	0/ −	0/ −	0/ −	0/0	*u*_3_/ −	${\bar{T}}_{33}/{\bar{S}}_{33}$
$C_{44}^{\text{eff}}$	*u*_3_/0	*u*_3_/0	0/ −	0/ −	*u*_1_/ −	*u*_1_/ −	${\bar{T}}_{13}/{\bar{S}}_{13}$
$C_{66}^{\text{eff}}$	*u*_2_/ −	*u*_2_/ −	*u*_1_/ −	*u*_1_/ −	0/0	0/0	${\bar{T}}_{12}/{\bar{S}}_{12}$
$e_{13}^{\text{eff}}$	0/ −	0/ −	0/ −	0/ −	0/0	0/Φ	$-{\bar{T}}_{11}/{\bar{E}}_{3}$
$e_{33}^{\text{eff}}$	0/ −	0/ −	0/ −	0/ −	0/0	0/Φ	$-{\bar{T}}_{33}/{\bar{E}}_{3}$
$e_{15}^{\text{eff}}$	*u*_3_/0	*u*_3_/0	0/ −	0/ −	*u*_1_/ −	*u*_1_/ −	${\bar{D}}_{1}/{\bar{S}}_{31}$
$\varepsilon_{11}^{\text{eff}}$	0/0	0/Φ	0/ −	0/ −	0/ −	0/ −	${\bar{D}}_{1}/{\bar{E}}_{1}$
$\varepsilon_{33}^{\text{eff}}$	0/ −	0/ −	0/ −	0/ −	0/0	0/Φ	${\bar{D}}_{3}/{\bar{E}}_{3}$

## 4 Results and Discussion

**Figure 3:**
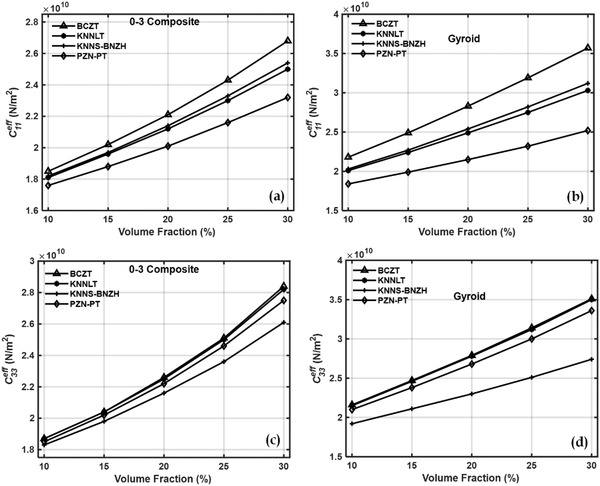
Effective elastic coefficient *C*_11_ of a) 0–3 piezocomposites, b) gyroid TPMS piezocomposites and $C_{33}^{\text{eff}}$ of c) 0–3 piezocomposites, d) gyroid TPMS piezocomposites.

**Figure 4:**
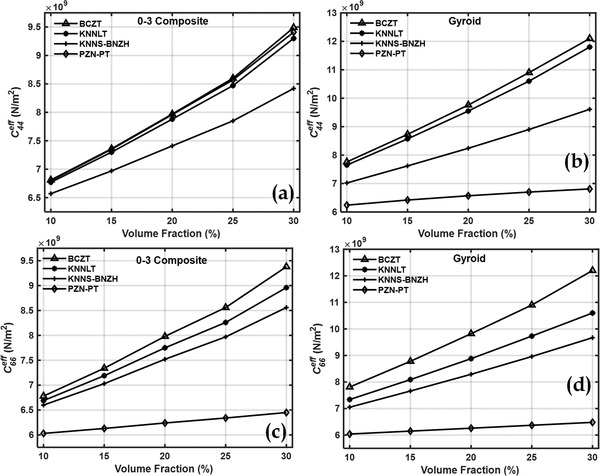
Effective elastic coefficient $C_{44}^{\text{eff}}$ of a) 0–3 piezocomposites, b) gyroid TPMS piezocomposites and $C_{66}^{\text{eff}}$ of c) 0–3 piezocomposites, d) gyroid TPMS piezocomposites.

Effective values of the elastic and piezoelectric properties of a cement matrix and piezoelectric inclusion‐based piezocomposite were calculated at all the different inclusion volume fractions for all the materials. **Figures** **3** and **4** show the effective elastic coefficients $C_{11}^{\text{eff}},C_{33}^{\text{eff}},C_{44}^{\text{eff}}$, and $C_{66}^{\text{eff}}$. Simple mixture rule explains the increase in effective properties with volume fractions of piezoelectric inclusions.

TPMS based piezocomposite demonstrated a significant increment in elastic properties compared to 0–3 composite. Stiffness coefficients increased by about 1.2 to 1.3 times in the case of gyroid TPMS piezocomposite. In the 0–3 composite, the inclusions are distributed randomly throughout the volume of the unit cell. Gyroid TPMS‐based piezocomposites, on the other hand, consist of mathematically defined surfaces.

TPMS piezoelectric inclusions, being mathematically defined surfaces, are symmetric and are a single structure as opposed to the random distribution of piezoelectric inclusions in the 0–3 composite. The single structure of the TPMS inclusion within the cement matrix makes them continuous and interconnected which could explain the higher values of the effective stiffness properties of the TPMS‐based piezocomposites compared to their 0–3 counterparts. This also makes them more robust compared to their 0–3 composites.^[^ ^80^, ^81^ ^]^ As TPMS‐based inclusions are mathematically defined continuous surfaces, the surfaces are much smoother leading to reduced surface roughness, which may explain the higher stiffness of the TPMS‐based piezocomposite material.^[^ ^23^ ^]^

**Figure 5:**
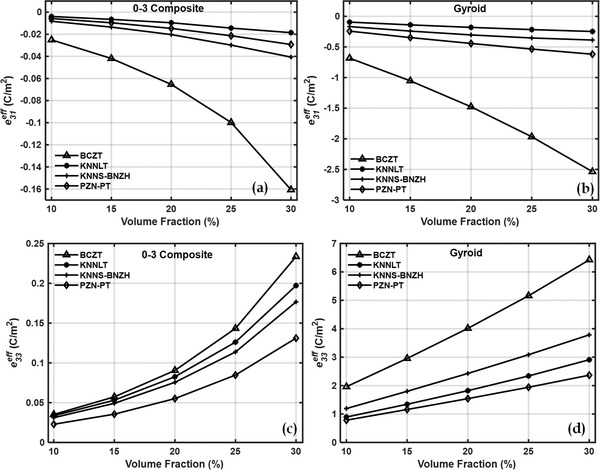
Effective piezoelectric coefficient $e_{13}^{\text{eff}}$ of a) 0–3 piezocomposites, b) gyroid piezocomposites and $e_{33}^{\text{eff}}$ of c) 0–3 piezocomposites, d) gyroid TPMS piezocomposites.

**Figure** **5** shows the effective piezoelectric properties of 0–3 composites and gyroid TPMS composites. For this case also simple mixture rule explains the increment of effective piezoelectric properties with volume fractions. A large enhancement in effective piezoelectric coefficient can be observed in gyroid inclusion‐based cement‐TPMS in comparison to 0–3 piezocomposite. At 30% volume fraction and for BCZT inclusion, the effective piezoelectric properties *e*_31_ and *e*_33_ of TPMS piezocomposite were found to be about 50 and 33 times higher than the corresponding 0–3 piezocomposite. This can also be attributed to better interconnectivity and continuity of matrix and inclusion material.^[^ ^80^, ^81^ ^]^ Continuity of the surface, interconnectivity and zero mean curvature of the interface between TPMS inclusion and the cement matrix are among the other significant reasons explaining the enhanced piezoelectric properties of the TPMS piezocomposites compared to its 0–3 counterparts.^[^ ^81^, ^82^ ^]^ It has already been reported in the literature that interconnectivity enhances different transport properties like thermal and electrical conductivity.^[^ ^81^, ^83^ ^]^ This probably could also explain the reason why TPMS‐based piezocomposites have enhanced piezoelectric properties than the 0–3 composite.

**Figure 6:**
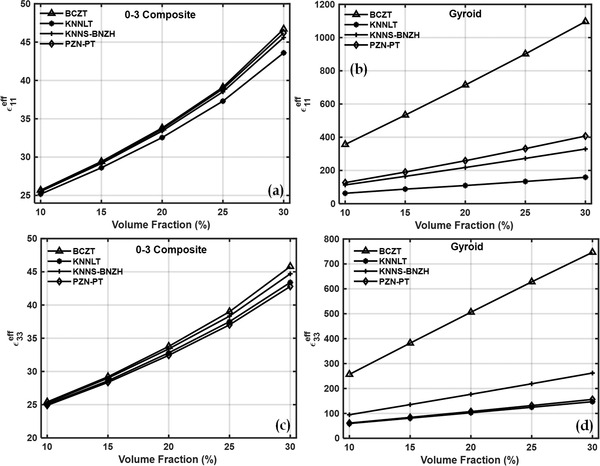
Effective relative permittivity $\varepsilon_{11}^{\text{eff}}$ of a) 0–3 piezocomposites, b) gyroid piezocomposites and $\varepsilon_{33}^{\text{eff}}$ of c) 0–3 piezocomposites and d) gyroid TPMS piezocomposites.

**Figure 7:**
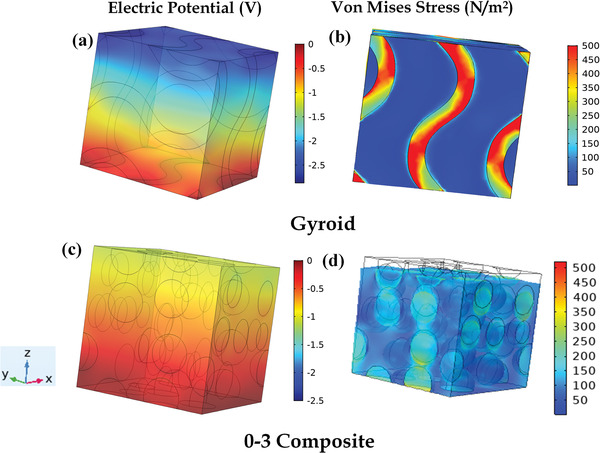
Voltage and Von Mises stress generated in response to a 100 N compressive load applied to the RVE of BCZT based piezoceramics at 30% volume fraction. a,b) Gyroid TPMS, c,d) 0–3 composite.

**Figure** **6** shows the effective relative permittivities of 0–3 and gyroid TPMS‐based piezocomposites. Effective relative permittivities of the cement‐BCZT composites at 30% volume fraction increased by about 15 to 20 times and can be explained by reasons discussed in the previous section. The above plots show that the BCZT inclusion‐based piezocomposite attains higher values of effective properties compared to other materials considered in the present study. **Figure** **7** shows the RVEs of the 0–3 and gyroid TPMS‐based piezocomposite at 30% volume fraction of inclusion, subject to an external compressive load of 100 N. In response to the external load of 100 N, voltage and stress increased by about 2.5 times and 2 times, respectively, when 0–3 composite is compared to gyroid TPMS composite. Surface continuity and zero mean curvature of the TPMS piezocomposite are the probable reasons which explain the higher stress‐bearing capacity and voltage of the TPMS piezocomposite than the 0–3 composite. Quantitatively speaking, the voltage is directly proportional to the piezoelectric coefficient and inversely to the relative permittivity. The larger increment in the magnitude of the piezoelectric coefficient compared to that of the relative permittivity in TPMS piezocomposite than in the 0–3 piezocomposite probably could explain the higher magnitude of the voltage response generated in TPMS RVE than in the 0–3 RVE.

Another important parameter used to quantify voltage response under force is piezoelectric voltage constant given by *g*_33_. The piezoelectric voltage constant is defined by Equation (13).^[^ ^84^ ^]^

(13) $$\begin{array}{c} \text{​}g_{33}=\frac{d_{33}}{\varepsilon_{33}^{T}} \end{array}$$

In Equation (13) *d*_33_ is the piezoelectric coefficient in strain charge form and $\varepsilon_{33}^{T}$ is the absolute permittivity of the material under constant stress (strain charge form). In the present paper, all the parameters were calculated in the stress–charge form, therefore to apply Equation (13), the strain charge piezoelectric parameters *d*_33_ and $\varepsilon_{33}^{T}$ are to be calculated from the stress charge piezoelectric parameters *e*_33_ and $\varepsilon_{33}^{S}$.

The stress–charge and strain–charge form of equations are given by Equations (14) to (17).^[^ ^85^ ^]^

Stress–charge form

(14) $$\begin{array}{c} T=c_{E}\times S-e^{t}\times E \end{array}$$

(15) $$\begin{array}{c} D=e\times S+\varepsilon_{S}\times E \end{array}$$

Strain charge form

(16) $$\begin{array}{c} S=s_{E}\times T+d^{t}\times E \end{array}$$

(17) $$\begin{array}{c} D=d\times T+\varepsilon_{T}\times E \end{array}$$

In Equations (14)–(17), **T** and **S** are stress and strain, respectively, **c**_**E**_ and **s**_**E**_ are stiffness and compliance coefficients, **e** ^*t*^ and **d** ^*t*^ are piezoelectric coefficients in stress and strain charge forms. ε_S_ and ε_T_ are permittivities in stress and strain–charge forms, respectively, and the terms **D** and **E** are electric displacement and electric field, respectively. Multiplying both sides of Equation (16) by $s_{E}^{-1}$ we get

(18) $$\begin{array}{c} s_{E}^{-1}\times S=T+s_{E}^{-1}\times d^{t}\times E \end{array}$$

Substituting **T** from Equation (14) into the above equation and using the mathematical relation that

(19) $$\begin{array}{c} {\left(AB\right)}^{t}=B^{t}A^{t} \end{array}$$

where *A*, *B* are matrices and *A^t^*,*B^t^* means transpose of the respective matrices, the relation between stress charge and strain–charge form of the piezoelectric coefficients can be calculated as

(20) $$\begin{array}{c} e=d\times s_{E}^{-1} \end{array}$$

Similarly, eliminating **D** from Equations (15) and (17) and using the relation

(21) $$\begin{array}{c} e=d\times s_{E}^{-1} \end{array}$$

the permittivity values in the stress charge and strain charge form are related to each other as

(22) $$\begin{array}{c} \varepsilon_{S}=\varepsilon_{T}-d\times s_{E}^{-1}\times d^{t} \end{array}$$

Using the above relations, the piezoelectric coefficients *d*_33_ in strain charge form for gyroid and 0–3 composites are calculated to be 2.6 × 10^−10^ C N^−1^ and 9.8 × 10^−12^ C N^−1^, respectively. Similarly, the permittivity in the strain charge forms can be calculated to be 8.8 × 10^−9^ and 4.1 × 10^−10^ F m^−1^, respectively. Correspondingly, the piezoelectric voltage constant *g*_33_ for gyroid and 0–3 piezocomposite are calculated to be *g*_33_ =  2.9 × 10^−2^ Vm N^−1^ for gyroid piezocomposite and *g*_33_ =  2.4 × 10^−2^ Vm N^−1^ for 0–3 piezocomposite. Comparing the piezoelectric voltage coefficient for both the gyroid and 0–3 piezocomposite it can be observed that the gyroid piezocomposite demonstrated about 21% increment in piezoelectric voltage constant *g*_33_ compared to 0–3 composite.

**Figure 8:**
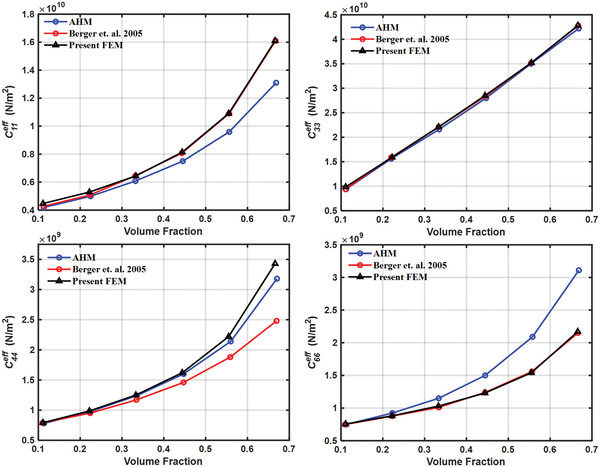
Validation of effective elastic properties.

**Figure 9:**
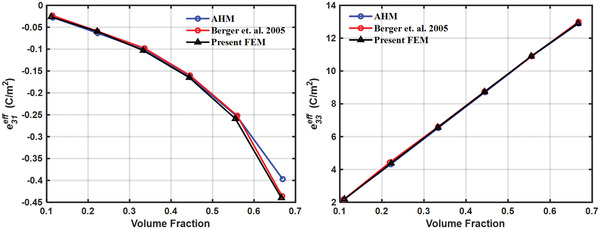
Validation of effective piezoelectric properties.

**Figure 10:**
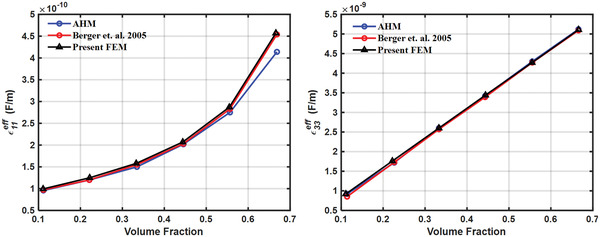
Validation of effective relative permittivity.

The finite element model used in the present study is validated by calculating the results obtained by Berger et al.^[^ ^71^ ^]^ for 1–3 piezocomposite made of lead zirconium titanate (PZT) as inclusion and a polymer matrix. The piezoelectric solid material considered is transversely isotropic while the polymer matrix material is considered to be isotropic material. The effective properties were calculated at different volume fractions by the finite element method, as well as, by the asymptotic homogenization method. AHM Volume fractions considered were 0.111, 0.222, 0.333, 0.444, 0.555, 0.666. The effective elastic and piezoelectric properties calculated by the present method are compared with those calculated by Berger et al. as shown in **Figures** 8, 9, 10.

## 5 Conclusion

Effective properties, both elastic and piezoelectric, of a cement‐based piezocomposite were calculated and studied for four different piezoelectric inclusion materials namely, BCZT,  KNNS − BNZH,  KNNLT, and PZN − PT at five different volume fractions of piezoelectric inclusions namely, 10% ,  15%,  20%,  25%,  and 30%. Effective properties were calculated by applying the finite element method to the RVE at different volume fractions. 0–3 piezocomposite and gyroid TPMS geometries were considered as inclusion geometries. Higher values of effective properties and voltages were observed for gyroid TPMS when compared to 0–3 piezocomposite. For BCZT material, at 30% volume fraction, the effective piezoelectric coefficients *e*_31_ and *e*_33_ increased by about 50 and 33 times and relative permittivity increased by about 15 to 20 times. In response to an external load of 100 N due to direct piezoelectric effect the voltage increased by about 2.5 times. The above study concludes that gyroid TPMS‐based piezocomposite with BCZT material inclusion can provide a better choice of material and inclusion type combination for a piezocomposite to obtain enhanced performance and which could be used for sensing applications in cement‐based buildings and structures. Sensing, actuation, and energy harvesting performance of the TPMS based piezocomposite can be studied in future studies.

## Conflict of Interest

The authors declare no conflict of interest.

## Data Availability

Data sharing is not applicable to this article as no new data were created or analyzed in this study.

## References

[1] K. S. Ramadan , D. Sameoto , S. Evoy , Smart Mater. Struct. 2014, 23, 033001.

[2] Z. Li , D. Zhang , K. Wu , J. Am. Ceram. Soc. 2002, 85, 305.

[3] P. Zhao , S. Kim , B. Hinderliter , J. Intell. Mater. Syst. Struct. 2016, 27, 1666.

[4] B. Dong , Z. Li , Compos. Sci. Technol. 2005, 65, 1363.

[5] Z. J. Li , D. Zhang , K. R. Wu , Mater. Struct. 2001, 34, 506.

[6] R. E. Newnham , A. Safari , J. Giniewicz , B. H. Fox , Ferroelectrics 1984, 60, 15.

[7] N. Jaitanong , R. Yimnirun , H. R. Zeng , G. R. Li , Q. R. Yin , A. Chaipanich , Mater. Lett. 2014, 130, 146.

[8] Z. Li , S. Huang , L. Qin , X. Cheng , Smart Mater. Struct. 2007, 16, 999.

[9] J. Sladek , P. Novak , P. L. Bishay , V. Sladek , Constr. Build. Mater. 2018, 190, 1208.

[10] R. E. Newnham , D. P. Skinner , L. E. Cross , Mater. Res. Bull. 1978, 13, 525.

[11] A. Safari , J. Phys. III France 1994, 4, 1129.

[12] J.‐H. Shin , S.‐H. Hong , J. Eur. Ceram. Soc. 2014, 34, 1297.

[13] D. W. Abueidda , R. K. A. Al‐Rub , A. S. Dalaq , D.‐W. Lee , K. A. Khan , I. Jasiuk , Mech. Mater. 2016, 95, 102.

[14] O. Al‐Ketan , R. K. A. Al‐Rub , Adv. Eng. Mater. 2019, 21, 1900524.

[15] O. Al‐Ketan , M. A. Assad , R. K. A. Al‐Rub , Compos. Struct. 2017, 176, 9.

[16] O. Al‐Ketan , et al., J. Therm. Sci. Eng. Appl. 2020, 13 https://asmedigitalcollection.asme.org/thermalscienceapplication/article/13/2/021010/1084162/Forced-Convection-Computational-Fluid-Dynamics.

[17] V. J. Challis , A. P. Roberts , A. H. Wilkins , Int. J. Solids Struct. 2008, 45, 4130.

[18] S. Torquato , A. Donev , Proc. R. Soc. A 2004, 460, 1849.

[19] S. Torquato , S. Hyun , A. Donev , Phys. Rev. Lett. 2002, 89, 266601. 12484843 10.1103/PhysRevLett.89.266601

[20] Z. Chen , Y. M. Xie , X. Wu , Z. Wang , Q. Li , S. Zhou , Mater. Des. 2019, 183, 108109.

[21] A. S. Dalaq , D. W. Abueidda , R. K. A. Al‐Rub , Composites, Part A 2016, 84, 266.

[22] A. S. Dalaq , D. W. Abueidda , R. K. A. Al‐Rub , I. M. Jasiuk , Int. J. Solids Struct. 2016, 83, 169.

[23] I. Maskery , L. Sturm , A. O. Aremu , A. Panesar , C. B. Williams , C. J. Tuck , R. D. Wildman , I. A. Ashcroft , R. J. M. Hague , Polymer 2018, 152, 62.

[24] E. Aksel , J. L. Jones , Sensors 2010, 10, 1935. 22294907 10.3390/s100301935 PMC3264460

[25] P. K. Panda , J. Mater. Sci. 2009, 44, 5049.

[26] H. Wei , H. Wang , Y. Xia , D. Cui , Y. Shi , M. Dong , C. Liu , T. Ding , J. Zhang , Y. Ma , N. Wang , Z. Wang , Y. Sun , R. Wei , Z. Guo , J. Mater. Chem. C 2018, 6, 12446.

[27] H. Xu , Y. M. Xie , R. Chan , S. Zhou , Compos. Sci. Technol. 2020, 200, 108417.

[28] X. Song , L. Yang , W. Wang , L. Chen , Proceedings of the ASME 2018 13th International Manufacturing Science and Engineering Conference Vol. 1, Texas, USA, 2018, 10.1115/MSEC2018-6704.

[29] D. Singh , R. Kiran , K. Chawla , R. Kumar , V. S. Chauhan , R. Vaish , Int. J. Eng. Sci. 2022, 178, 103726.

[30] S. Karmakar , R. Kiran , R. Vaish , V. S. Chauhan , J. Intell. Mater. Syst. Struct. 2022, 33, 1929.

[31] B. Han , L. Zhang , J. Ou , in Self‐Compacting Concrete BT—Smart and Multifunctional Concrete Toward Sustainable Infrastructures (Eds: B. Han , L. Zhang , J. Ou ), Springer Singapore, Singapore 2017, pp. 11–36.

[32] B. Han , L. Zhang , J. Ou , in Self‐Expanding Concrete BT—Smart and Multifunctional Concrete Toward Sustainable Infrastructures (Eds: B. Han , L. Zhang , J. Ou ), Springer Singapore, Singapore 2017, pp. 37–53.

[33] B. Han , L. Zhang , J. Ou , in Self‐Curing Concrete BT—Smart and Multifunctional Concrete Toward Sustainable Infrastructures (Eds: B. Han , L. Zhang , J. Ou ), Springer Singapore, Singapore 2017, pp. 55–66.

[34] B. Han , L. Zhang , J. Ou , in Self‐Shaping Concrete BT—Smart and Multifunctional Concrete Toward Sustainable Infrastructures (Eds: B. Han , L. Zhang , J. Ou ), Springer Singapore, Singapore 2017, pp. 67–80.

[35] B. Han , L. Zhang , J. Ou , in Self‐Sensing Concrete BT—Smart and Multifunctional Concrete Toward Sustainable Infrastructures (Eds: B. Han , L. Zhang , J. Ou ), Springer Singapore, Singapore 2017, p. 81.

[36] B. Han , L. Zhang , J. Ou , in Self‐Healing Concrete BT—Smart and Multifunctional Concrete Toward Sustainable Infrastructures (Eds: B. Han , L. Zhang , J. Ou ), Springer Singapore, Singapore 2017, p. 117.

[37] B. Han , L. Zhang , J. Ou , in Self‐Adjusting Concrete BT—Smart and Multifunctional Concrete Toward Sustainable Infrastructures (Eds: B. Han , L. Zhang , J. Ou ), Springer Singapore, Singapore 2017, p. 157.

[38] B. Han , L. Zhang , J. Ou , in Damping Concrete BT—Smart and Multifunctional Concrete Toward Sustainable Infrastructures (Eds: B. Han , L. Zhang , J. Ou ), Springer Singapore, Singapore 2017, p. 177.

[39] B. Han , L. Zhang , J. Ou , in Anti‐Spalling Concrete BT—Smart and Multifunctional Concrete Toward Sustainable Infrastructures (Eds: B. Han , L. Zhang , J. Ou ), Springer Singapore, Singapore 2017, p. 191.

[40] B. Han , L. Zhang , J. Ou , in Wear‐Resisting Concrete BT—Smart and Multifunctional Concrete Toward Sustainable Infrastructures (Eds: B. Han , L. Zhang , J. Ou ), Springer Singapore, Singapore 2017, p. 223.

[41] B. Han , L. Zhang , J. Ou , in Aircraft Arresting Concrete BT—Smart and Multifunctional Concrete Toward Sustainable Infrastructures (Eds: B. Han , L. Zhang , J. Ou ), Springer Singapore, Singapore 2017, p. 235.

[42] B. Han , L. Zhang , J. Ou , in Electrically Conductive Concrete BT—Smart and Multifunctional Concrete Toward Sustainable Infrastructures (Eds: B. Han , L. Zhang , J. Ou ), Springer Singapore, Singapore 2017, p. 247.

[43] B. Han , L. Zhang , J. Ou , in Electrothermal Concrete BT—Smart and Multifunctional Concrete Toward Sustainable Infrastructures (Eds: B. Han , L. Zhang , J. Ou ), Springer Singapore, Singapore 2017, p. 261.

[44] B. Han , L. Zhang , J. Ou , in Light‐Transmitting Concrete BT—Smart and Multifunctional Concrete Toward Sustainable Infrastructures (Eds: B. Han , L. Zhang , J. Ou ), Springer Singapore, Singapore 2017, p. 273.

[45] B. Han , L. Zhang , J. Ou , in Light‐Emitting Concrete BT—Smart and Multifunctional Concrete Toward Sustainable Infrastructures (Eds: B. Han , L. Zhang , J. Ou ), Springer Singapore, Singapore 2017, p. 285.

[46] B. Han , L. Zhang , J. Ou , in Photocatalytic Concrete BT—Smart and Multifunctional Concrete Toward Sustainable Infrastructures (Eds: B. Han , L. Zhang , J. Ou ), Springer Singapore, Singapore 2017, p. 299.

[47] B. Han , L. Zhang , J. Ou , in Electromagnetic Wave Shielding/Absorbing Concrete BT—Smart and Multifunctional Concrete Toward Sustainable Infrastructures (Eds: B. Han , L. Zhang , J. Ou ), Springer Singapore, Singapore 2017, p. 313.

[48] B. Han , L. Zhang , J. Ou , in Radiation Shielding Concrete BT—Smart and Multifunctional Concrete Toward Sustainable Infrastructures (Eds: B. Han , L. Zhang , J. Ou ), Springer Singapore, Singapore 2017, p. 329.

[49] B. Han , L. Zhang , J. Ou , in Energy‐Harvesting Concrete BT—Smart and Multifunctional Concrete Toward Sustainable Infrastructures (Eds: B. Han , L. Zhang , J. Ou ), Springer Singapore, Singapore 2017, p. 379.

[50] B. Han , S. Ding , X. Yu , Measurement 2015, 59, 110.

[51] B. Han , Y. Wang , S. Dong , L. Zhang , S. Ding , X. Yu , J. Ou , J. Intell. Mater. Syst. Struct. 2015, 26, 1303.

[52] X. Wang , S. Dong , A. Ashour , B. Han , J. Mater. Sci. 2021, 56, 16243.

[53] P. Kubala , P. Batys , J. Barbasz , P. Weroński , M. Cieśla , Adv. Colloid Interface Sci. 2022, 306, 102692. 35753239 10.1016/j.cis.2022.102692

[54] J.‐S. Wang , Int. J. Mod. Phys. C 1994, 05, 707.

[55] J. Zhou , L. Qi , A. M. Gokhale , J. Eng. Mater. Technol. 2016, 138, 021001.

[56] Y. Zhang , M. Xie , J. Roscow , C. Bowen , Mater. Res. Bull. 2019, 112, 426.

[57] F.‐Z. Yao , K. Wang , J.‐F. Li , J. Appl. Phys. 2013, 113, 174105.

[58] L. Qiao , G. Li , H. Tao , J. Wu , Z. Xu , F. Li , Ceram. Int. 2020, 46, 5641.

[59] J. Jin , K. K. Rajan , L. C. Lim , Jpn. J. Appl. Phys. 2006, 45, 8744.

[60] H. L. W. Chan , J. Unsworth , IEEE Trans. Ultrason., Ferroelectr., Freq. Control 1989, 36, 434. 18285003 10.1109/58.31780

[61] W. A. Smith , B. A. Auld , IEEE Trans. Ultrason., Ferroelectr., Freq. Control 1991, 38, 40. 18267555 10.1109/58.67833

[62] P. Gaudenzi , Comput. Struct. 1997, 65, 157.

[63] M. Melnykowycz , X. Kornmann , C. Huber , M. Barbezat , A. J. Brunner , Smart Mater. Struct. 2006, 15, 204.

[64] Y. Benveniste , J. Appl. Phys. 1992, 72, 1086.

[65] M. L. Dunn , M. Taya , Int. J. Solids Struct. 1993, 30, 161.

[66] T. Chen , J. Mech. Phys. Solids 1993, 41, 1781.

[67] W. Biao , Int. J. Solids Struct. 1992, 29, 293.

[68] J. Y. Li , M. L. Dunn , Philos. Mag. A 2001, 81, 903.

[69] J. L. Teply , G. J. Dvorak , J. Mech. Phys. Solids 1988, 36, 29.

[70] C. Poizat , M. Sester , Comput. Mater. Sci. 1999, 16, 89.

[71] H. Berger , S. Kari , U. Gabbert , R. Rodriguez‐Ramos , R. Guinovart , J. A. Otero , J. Bravo‐Castillero , Int. J. Solids Struct. 2005, 42, 5692.

[72] H. Berger , S. Kari , U. Gabbert , R. Rodriguez‐Ramos , J. Bravo‐Castillero , R. Guinovart‐Diaz , F. J. Sabina , G. A. Maugin , Smart Mater. Struct. 2006, 15, 451.

[73] S. Nemat‐Nasser , M. Lori , S. K. Datta , J. Appl. Mech. 1996, 63, 561.

[74] N. Yang , Z. Quan , D. Zhang , Y. Tian , CAD Comput. Aided Des. 2014, 56, 11.

[75] K. A. Brakke , Exp. Math. 1992, 1, 141.

[76] D.‐J. Yoo , Int. J. Precis. Eng. Manuf. 2011, 12, 61.

[77] M.‐T. Hsieh , L. Valdevit , Software Impacts 2020, 6, 100026.

[78] O. Al‐Ketan , R. K. A. Al‐Rub , Mater. Des. Process. Commun. 2021, 3, e205.

[79] E. L. C. N. Silva , J. S. O. Fonseca , N. Kikuchi , Comput. Methods Appl. Mech. Eng. 1998, 159, 49.

[80] D. W. Abueidda , M. Bakir , R. K. A. Al‐Rub , J. S. Bergström , N. A. Sobh , I. Jasiuk , Mater. Des. 2017, 122, 255.

[81] D. W. Abueidda , A. S. Dalaq , R. K. A. Al‐Rub , H. A. Younes , Int. J. Mech. Sci. 2015, 92, 80.

[82] H.‐Y. Chen , Y. Kwon , K. Thornton , Scr. Mater. 2009, 61, 52.

[83] Z. Poniznik , V. Salit , M. Basista , D. Gross , Comput. Mater. Sci. 2008, 44, 813.

[84] I. Mahmud , S.‐C. Ur , M.‐S. Yoon , J. Korean Phys. Soc. 2014, 65, 133.

[85] A. Erturk , D. J. Inman , in Piezoelectric Energy Harvesting, (Eds: A. Erturk , D. J. Inman ) John Wiley & Sons, Ltd, 2011 p. 343.

